# Subcellular Localization of Thioredoxin/Thioredoxin Reductase System—A Missing Link in Endoplasmic Reticulum Redox Balance

**DOI:** 10.3390/ijms25126647

**Published:** 2024-06-17

**Authors:** Krisztina Veszelyi, Ibolya Czegle, Viola Varga, Csilla Emese Németh, Balázs Besztercei, Éva Margittai

**Affiliations:** 1 Institute of Translational Medicine, Semmelweis University, H-1085 Budapest, Hungary; veszelyi.krisztina@phd.semmelweis.hu (K.V.); varviola@gmail.com (V.V.); besztercei.balazs@semmelweis.hu (B.B.); 2 Department of Internal Medicine and Haematology, Semmelweis University, H-1085 Budapest, Hungary; czibolyka@gmail.com; 3 Institute of Biochemistry and Molecular Biology, Department of Molecular Biology, Semmelweis University, H-1085 Budapest, Hungary; nemeth.csilla@med.semmelweis-univ.hu

**Keywords:** endoplasmic reticulum, thioredoxin/thioredoxin reductase, redox homeostasis, subcellular distribution

## Abstract

The lumen of the endoplasmic reticulum (ER) is usually considered an oxidative environment; however, oxidized thiol-disulfides and reduced pyridine nucleotides occur there parallelly, indicating that the ER lumen lacks components which connect the two systems. Here, we investigated the luminal presence of the thioredoxin (Trx)/thioredoxin reductase (TrxR) proteins, capable of linking the protein thiol and pyridine nucleotide pools in different compartments. It was shown that specific activity of TrxR in the ER is undetectable, whereas higher activities were measured in the cytoplasm and mitochondria. None of the Trx/TrxR isoforms were expressed in the ER by Western blot analysis. Co-localization studies of various isoforms of Trx and TrxR with ER marker Grp94 by immunofluorescent analysis further confirmed their absence from the lumen. The probability of luminal localization of each isoform was also predicted to be very low by several in silico analysis tools. ER-targeted transient transfection of HeLa cells with Trx1 and TrxR1 significantly decreased cell viability and induced apoptotic cell death. In conclusion, the absence of this electron transfer chain may explain the uncoupling of the redox systems in the ER lumen, allowing parallel presence of a reduced pyridine nucleotide and a probably oxidized protein pool necessary for cellular viability.

## 1. Introduction

Endoplasmic reticulum (ER) is an elaborated membrane network in the cytosol of most eukaryotic cells, and its lumen is considered as an individual organelle with a vast spectrum of unique functions. The lumen is enclosed by membranes possessing selective permeability, allowing the ER to maintain its own proteome, metabolome, and specific intraluminal reactions, including those that maintain the redox environment. Principal redox active components of the ER are similar to the redox machineries of other intracellular compartments; however, their concentrations and redox states may vary significantly between organelles.

Redox systems in the ER lumen constitute a special, two-center based network. The first system, responsible for biosynthesis, biotransformation, and antioxidant defense, is organized around pyridine nucleotides. NADPH is produced by the hexose-6-phosphate dehydrogenase (H6PDH) within the lumen and contributes with a yet-unknown mechanism to the antioxidant defense of the lumen. Furthermore, it is a cofactor of the 11β-Hydroxysteroid dehydrogenase type 1 (11βHSD1) oxidoreductase of the ER, which can catalyze the luminal reduction of cortisone to cortisol [1]. Luminal NADPH may also be necessary for adrenal CytP450-related steroidogenic pathways [2] and for NAD(P)H cytochrome b5 oxidoreductase (Ncb5or) [3]. Since the ER membrane is impermeable to the pyridine nucleotides, these enzymes must use a separate, luminally located NADP(H) pool [4]. Investigation of the redox state showed that pyridine nucleotides are predominantly reduced in the lumen, thus being able to ensure the abovementioned processes.

The second redox system is involved in the post-translational modification of secretory proteins and utilizes glutathione (GSH)/glutathione disulfide (GSSG), ascorbate/dehydroascorbic acid, and vitamin K. The central enzyme of the second system is the protein disulfide isomerase (PDI), which participates in the processes of oxidative folding, vitamin K cycle, and dehydroascorbic acid reduction. Thus, electrons derived from the reaction of disulfide bond formation can be used by this mechanism to regenerate active vitamin K and ascorbate [5]. Conventionally, the luminal space has been characterized as more oxidizing than the cytosol due to the presence of the oxidative protein folding: proteins synthesized and processed here have remarkably more disulfide bridges and less free cysteinyl thiols than the cytosolic ones. This phenomenon is reflected in the altered ratio of glutathione to glutathione disulfide (i.e., the luminal [GSH]:[GSSG] ratio is nearly 20 times lower than in the cytosol) [6,7,8].

Colocalized redox pairs are usually linked by oxidoreductases to form a more sophisticated, complex redox system. However, the lack of linking enzymes, and the uncoupled state of these two intraluminal redox systems in the ER, have been previously implicated, mainly because of the coexistence of a reduced pyridine nucleotide and an oxidized protein/glutathione pool [5,9,10]. If the linking enzymes are missing in the ER lumen, the redox pairs can coexist independently and have different redox potentials: despite the oxidizing power of the GSSG/GSH system, pyridine nucleotides may remain reduced. The fact that the uncoupling of the thiol/disulfide and NAD(P)H/NAD(P)^+^ redox couples occurred as a result of their subcompartmentation was excluded: these two main redox systems in the ER—the thiol/disulfide and the pyridine nucleotide systems—are not isolated from each other within the compartment [9] but are distributed in all subfractions of the lumen.

Glutathione reductase and thioredoxin reductases (TrxRs) are key factors that catalyze electron transfer and maintain the connection between reduced pyridine nucleotides and glutathione disulfide. Glutathione reductase is well represented in the cytosol and in the mitochondria; however, there are only sporadic reports of its occurrence in the ER. A 10-fold lower glutathione reductase activity was reported in rat liver microsomal vesicles than in the cytosol [11]. Previous results of our laboratory also suggest that the expression and activity of glutathione reductase are practically absent in rat liver microsomal vesicles [9]. Like glutathione reductase, TrxRs can maintain a possible electron flux between the two redox systems via the conversion of thioredoxin (Trx) from an oxidized to a reduced form using NAPDH [12]. TrxRs play a crucial role in the antioxidant process, regulation of intracellular redox potential, and programmed cell death [12,13,14]. Thioredoxins, with a dithiol/disulfide active site (CGPC), are the main cellular protein disulfide reductases; therefore, they also serve as electron donors for ribonucleotide reductases, thioredoxin peroxidases (peroxiredoxins), and methionine sulfoxide reductases. Fundamentally, the thioredoxin reductase enzyme has three distinct isoforms (TrxR1, TrxR2, and TrxR3) that are present in various mammalian tissues and subcellular compartments of the mammalian cells. While TrxR1 is suggested to be present in the cytoplasm, TrxR2 is considered to be the mitochondrial isoform [15,16]. The cytosolic Trx1/TrxR1 system is involved in the regulation of transcription factors, protein repair, and apoptosis. Due to its antioxidant activity, Trx1 is able to protect against oxidative stress when upregulated or overexpressed [17]. The mitochondrial Trx2/TrxR2 system is also suggested to be involved in the regulation of transcription factors and has several other roles, e.g., the regulation of mitochondria-driven cell death, the mitochondrial integrity, detoxification of aldehydes, protein synthesis and folding, and metabolic processes [18]. Interestingly, overexpression of Trx2 increased the production of mitochondrial reactive oxygen species (ROS) in hypoxia [19]. Trx1 and Trx2 may respond differently to particular changes in the redox state of the cell. Increased ROS production upon the addition of epidermal growth factor caused a selective oxidation of cytoplasmic Trx1. On the other hand, a preferential oxidation of Trx2 was observed upon tumor necrosis factor α (TNFα) treatment [18].

The importance of the presence and localization of the Trx/TrxR system emerged by recent findings that the isoforms have been associated with a wide variety of human diseases (reviewed in [20]). The Trx/TrxR system plays a crucial role in the physiology of the adipose tissue, namely in its carbohydrate metabolism, insulin production and sensitivity, blood pressure regulation, inflammation, chemotactic activity of macrophages, and atherogenesis. Interestingly, current evidence suggests that the modulation of the Trx/TrxR system may be a novel target in the management of the metabolic syndrome, insulin resistance, and type 2 diabetes [21], as high levels of the Trx1 protein suppress the progression of the disease. It ameliorates glucose intolerance and enhances and preserves beta-cell functions, especially the insulin-secreting capacity [22]. In cardiovascular disease, Trx1 has an impact on atherosclerosis via influencing the NO system [23] and also plays a role in cardiac hypertrophy [24], heart failure, and myocarditis [25]. In the nervous system, the acquired or genetic dysfunction of Trx or TrxR could predispose neurons for degeneration [26], as observed in Alzheimer’s or Parkinson’s disease.

Furthermore, elevated TrxR level and activity have also been described in numerous cancer cells, making the system a new potential therapeutic target in oncological treatments [27,28,29,30].

In order to elucidate the pathophysiological role of this protein family in human diseases and to exploit them as possible therapeutic targets, it is crucial to understand their intracellular localization and their contribution to the maintenance of subcellular redox homeostasis.

Only one study from almost 30 years ago suggested that thioredoxins might be absent from the ER [31]; however, an extensive analysis of the subcellular localization of the proteins is still missing. According to our hypothesis, similar to glutathione reductase, thioredoxin reductases might be absent from the lumen of the ER. In this article, we further investigated the localization and the potential absence of Trx/TrxR enzymes as one of the essential reasons for the uncoupled redox systems in the ER.

## 2. Results

### 2.1. In Silico Analysis of Trx/TrxR Localization with Different Prediction Tools

To confirm the luminal absence of Trx and TrxR isoforms, in silico analysis was performed with eight subcellular localization prediction programs (Table 1). The presented data show the predicted probability of ER localization of each Trx/TrxR isoform. In summary, a low probability of ER localization was found for all Trx/TrxR isoforms using each prediction program.

PSORT II analyzes the features of the protein sequence (e.g., sorting signals, motifs) that influence the intracellular localization and subsequently estimates the probability of the protein being present in each localization site, while displaying the most likely localization [32].

Predotar is a neural network-based approach that uses the charge and the hydrophobicity of amino acid side chains to predict subcellular localization. The output localization represents the probability that the input sequence contained the given target signal. In case of the ER, the hydrophobic target signal located 15–30 residues from the N-terminal is the major determinant recognized by Predotar [33].

Cello uses an approach based on a two-level support vector machine (SVM) system, and the location with the largest displayed probability is used as the prediction. CELLO II performs especially well for the cytoplasmic localization, but it is not as accurate in detecting other subcellular localizations [34].

Multiloc also uses an SVM-based approach, which integrates N-terminal targeting sequences, amino acid composition, and protein sequence motifs to predict the intracellular location of the protein [35].

YLoc-HighRes was trained on the Höglund dataset and derived about 30,000 features from the protein sequence using amino acid composition, pseudo composition, and physical properties such as hydrophobicity, charge, and volume. PROSITE motifs and GO terms from close homologs are also included in the predictions [36].

LocTree3 combines the machine learning-based LocTree2 and a homology-based interference by a BLAST search of proteins with known subcellular location [37].

DeepLoc uses deep neural networks to predict protein subcellular localization, which takes into account the entire protein sequence with an attention mechanism identifying protein regions important for subcellular localization. The model was trained and tested on a protein dataset extracted from one of the latest UniProt releases, in which experimentally annotated proteins follow more stringent criteria than before. DeepLoc-1.0 ’s networks operate with the entire protein sequence and their corresponding location labels, but it does not recognize sorting signals separately [38].

We further examined the likelihood of the occurrence of thioredoxin reductase 3 (TrxR3) in certain subcellular compartments by using the same prediction tools (Table 2), since its location is the least studied among the Trx/TrxR isoforms. All the prediction tools used uniformly showed the highest probability of cytoplasmic localization of TrxR3, while its ER localization was unlikely according to the programs used.

### 2.2. Activity of TrxR in Subcellular Compartments of Rat Liver

To investigate the presence of TrxRs in subcellular organelles, enzyme activity was measured in various subcellular compartments isolated from rat liver. We found that TrxRs showed high specific activities in the mitochondrial (1.57 U/mg ± 0.19) and in the cytoplasmic fractions (1.26 U/mg ± 0.11). As expected, the specific activity of TrxRs in the ER fraction was undetectable (0.02 U/mg ± 0.01), and the optical density (OD, reflecting the amount of the reaction product catalyzed by TrxR) remained unchanged over time, indicating the absence of TrxR activity from the ER. 

**Figure 1:**
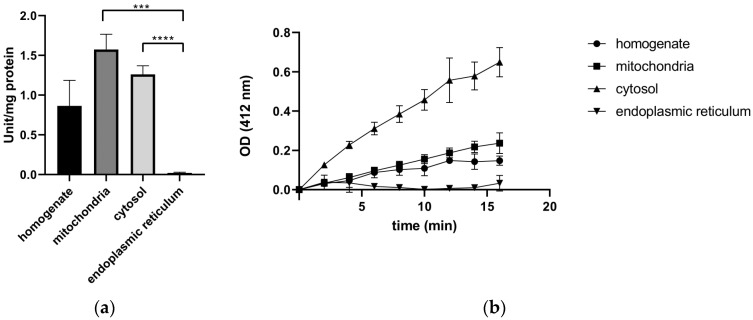
Thioredoxin reductase activity is absent from the endoplasmic reticulum. Thioredoxin reductase activity was measured colorimetrically with the Thioredoxin Reductase Assay Kit in various subcellular fractions isolated from rat liver (homogenate, mitochondria, cytosol, and the endoplasmic reticulum fraction). (**a**) Specific activity of TrxR in each subcellular fraction; *p* values: ***: 0.0002, **** = <0.0001, *t*-test. (**b**) Time dependence of optical density (OD, corresponding to the amount of the reaction product catalyzed by TrxR).

Finally, the highest specific activity of TrxRs appeared in the cytoplasmic and in the mitochondrial fractions (Figure 1a), indicating the apparent cytosolic and mitochondrial localization of the TrxRs. Cytosolic TrxR activity is presumably due to TrxR1, whereas the mitochondrial activity might be mainly attributed to TrxR2. The higher specific activity of the mitochondrial fraction compared to the cytosol is due to the differences in protein concentration of the organelles. In the homogenate, lower activity was measured due to its relatively lower TrxR concentration. Finally, the nearly undetectable specific activity of TrxRs in the endoplasmic reticulum fraction (Figure 1a) and their persistently low absorbance (Figure 1b) support the assumption of the lack of TrxRs in the endoplasmic reticulum of liver cells.

### 2.3. Expression of Trx/TrxR Isoforms in Subcellular Compartments of Rat Liver

**Figure 2:**
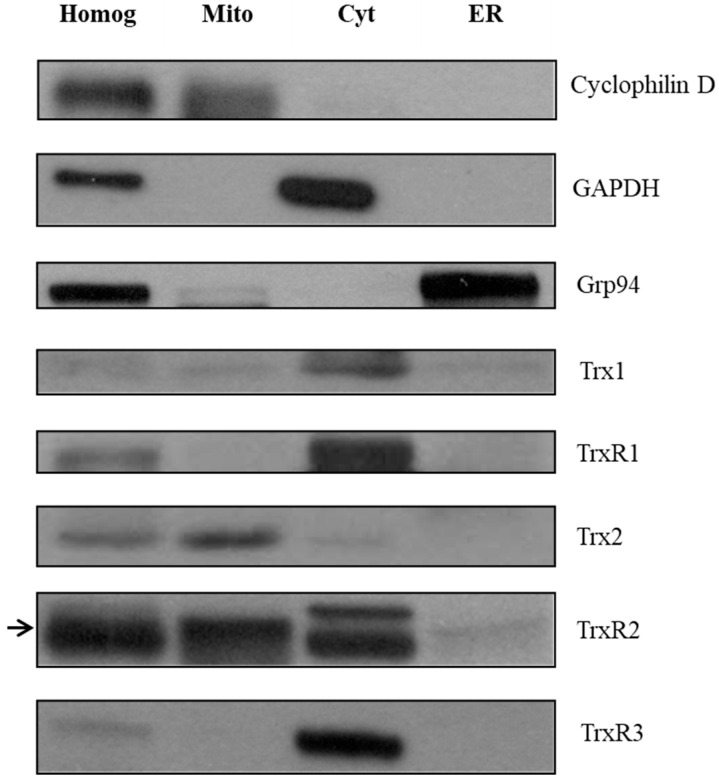
Thioredoxin and thioredoxin reductase isoforms are not localized in the endoplasmic reticulum fraction of rat liver. The expression of different Trx and TrxR isoforms was investigated with Western blot after SDS-PAGE separation of proteins, on purified subcellular fractions (homogenate, mitochondria, cytosol, and the ER) of rat liver. The purity of the fractions was tested with organelle-specific markers, i.e., Cyclophilin D for mitochondria, GAPDH for cytosol, and Grp94 for ER. The expression of each Trx and TrxR isoform was analyzed on the subcellular fractions. Equal amounts of protein were loaded in each lane (25 μg). Homog, homogenate; Mito, mitochondria; Cyt, cytosol; ER, endoplasmic reticulum. The arrow on the left indicates the bands corresponding to TrxR2.

We aimed to further confirm the localization of Trxs and TrxRs in rat liver subcellular fractions by Western blot analysis (Figure 2). First, the purity values of the fractions were verified by organelle-specific marker proteins (Cyclophilin D for mitochondria, GAPDH for cytosol and Grp94 for microsomal fraction). Secondly, the Trx1 (12 kDa), Trx2 (13 kDa), TrxR1 (55 kDa), TrxR2 (56 kD) and TrxR3 (65 kDa) proteins were decorated with their specific antibodies in each fraction. According to previous data [39], cytosolic location of Trx1 and TrxR1 were observed. In addition, Trx2 and TrxR2 showed mitochondrial localization, which is consistent with our current knowledge [40,41]. Finally, TrxR3, which has the most uncertain localization based on previous studies, appeared in the cytosolic fraction. In conclusion, none of the examined Trxs and TrxRs were localized in the endoplasmic reticulum fraction.

### 2.4. None of the Trx/TrxR Isoforms Co-Localizes with ER Marker Protein Grp94

**Figure 3:**
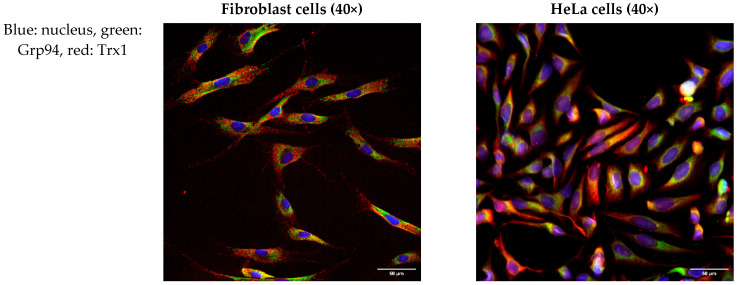

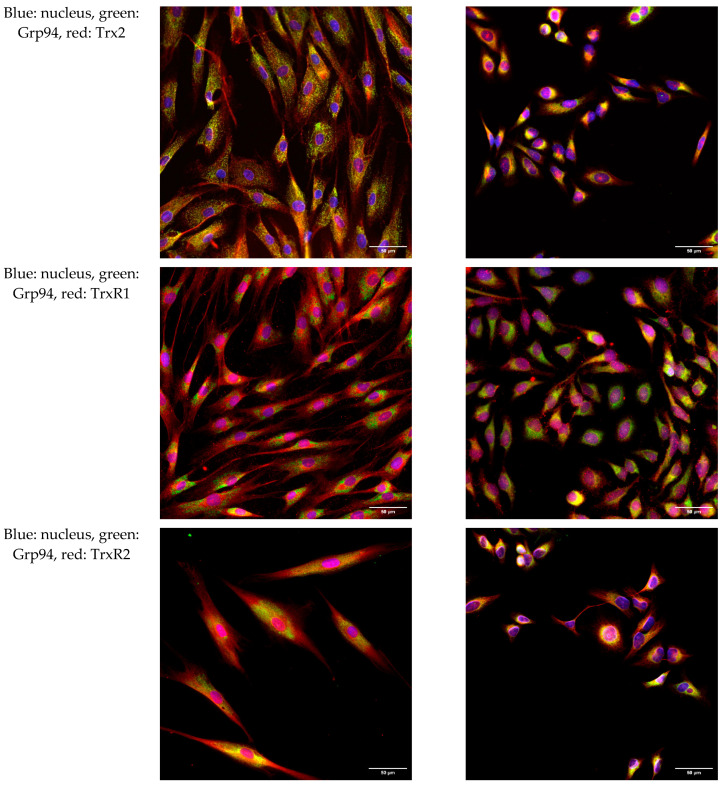

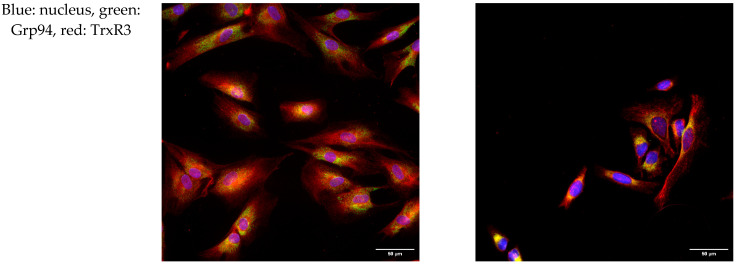
Trx/TrxR isoforms do not show co-localization with the endoplasmic reticulum marker Grp94. hTERT-immortalized human fibroblast and HeLa cells were immunoreacted with antibodies to different Trx/TrxR isoforms and the endoplasmic reticulum (ER) marker Grp94, as described in the Materials and Methods section. The images were acquired by fluorescent microscopy, as reported in the Materials and Methods section. Images were acquired with 40× magnification. Scalebar: 50 µm.

To further investigate the intracellular localization of Trx/TrxR isoforms, immunofluorescence analysis was performed on hTERT-immortalized human fibroblasts and HeLa cells. ER was labeled with the Grp94 marker protein, and possible co-localization was studied with Trx/TrxR isoforms. Our results show that none of the isoforms show evident co-localization with Grp94 (Figure 3), indicating the luminal absence of Trx/TrxR.

**Figure 4:**
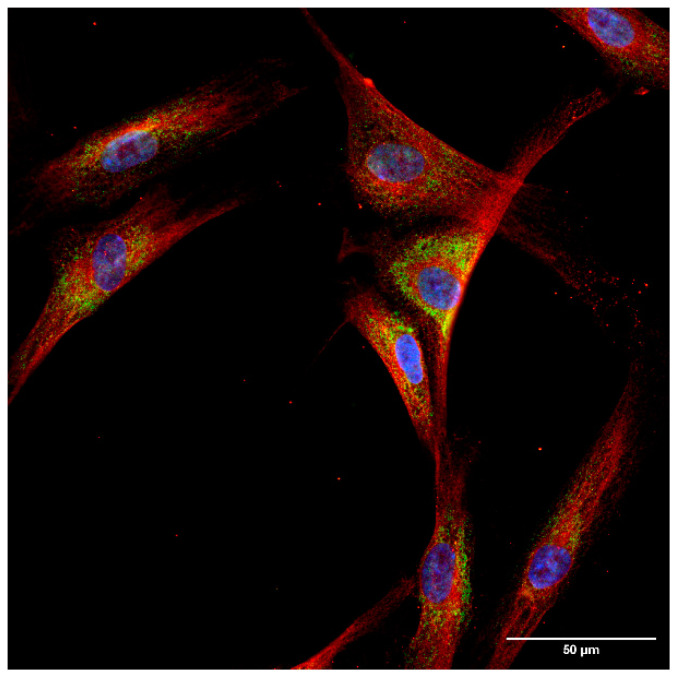
Thioredoxin reductase 3 is localized in the cytosol of hTERT-immortalized human fibroblast cells. Fibroblast cells were immunoreacted with antibodies against TrxR3 and the endoplasmic reticulum marker Grp94, as described in the Materials and Methods section. Images were acquired by fluorescence microscopy, as reported in the Materials and Methods. Images were obtained at 60× magnification. Blue: nucleus, green: Grp94, red: TrxR3. Scalebar: 50 µm.

**Figure 5:**
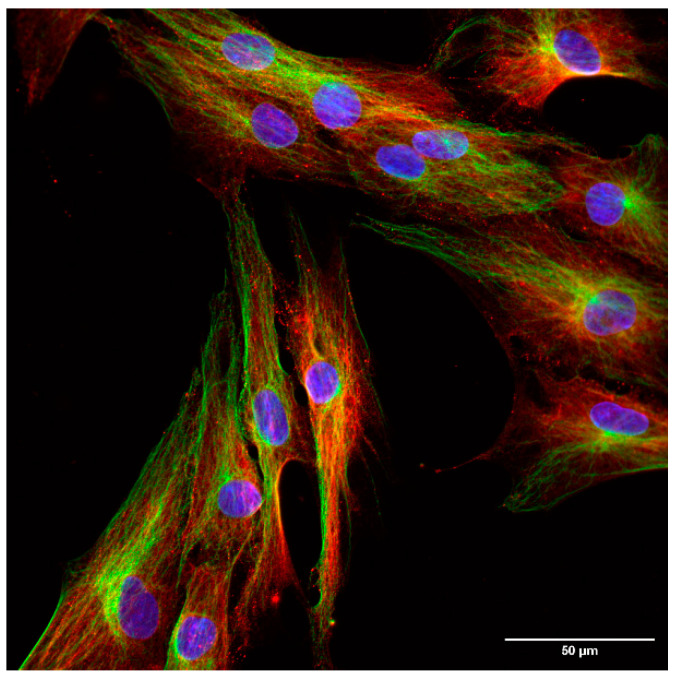
Immunofluorescent analysis of hTERT-immortalized human fibroblast cells (60×). Blue: nucleus, green: tubulin, red: TrxR3. Scalebar: 50 µm.

The localization of the least examined isoform, TrxR3, was addressed in a separate experimental series to extensively analyze its subcellular distribution on fibroblast cells. A clearly distinct, non-overlapping localization of TrxR3 and the ER-specific protein, Grp94 was observed (Figure 4). In order to better visualize the cellular shape, cells were immunoreacted with an antibody against a cytoskeleton-specific protein, tubulin (Figure 5). Although tubulin and TrxR3 did not show co-localization, the intracellular distribution of the signal corresponding to TrxR3 strongly implies its cytosolic distribution.

### 2.5. ER-Targeted Expression of Trx1/TrxR1 in HeLa Cells Severely Compromised Cell Viability and Induced Apoptotic Cell Death

After showing that the ER lacks Trx/TrxR redox systems, we were interested to see the consequences of co-expressing Trx1 and TrxR1 in the lumen of the ER, which would be able to artificially connect reduced pyridine nucleotides with oxidized proteins. 

**Figure 6:**
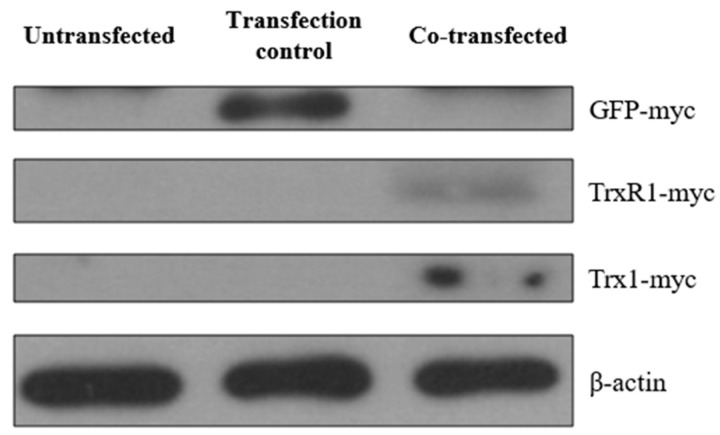
Expression of myc-tagged GFP, Trx1, and TrxR1 in transfected HeLa cells. Cell lysates from un-transfected HeLa cells, pCMV-ER/GFP-myc plasmid transfected cells, and pCMV-ER/Trx1-myc and pCMV-ER/TrxR1-myc co-transfected HeLa cells were analyzed by Western blot analysis after SDS-PAGE separation of proteins. The myc-tagged proteins were probed with anti-myc antibody to confirm the expression of pCMV-ER/GFP-myc in the transfection control, and pCMV-ER/Trx1-myc and pCMV-ER/TrxR1-myc in the co-transfected cells. The myc-tag adds approximately 2 kDa to the original molecular weight of the protein; therefore, GFP-myc (29 kDa), TrxR1-myc (57 kDa), and Trx1-myc (14 kDa) were detected according to their molecular weight. Equal amounts of protein were loaded in each lane (25 μg). β-actin was used as a loading control.

For this purpose, HeLa cells were co-transfected with pCMV-ER/Trx1-myc and pCMV-ER/TrxR1-myc plasmids containing an ER signal sequence and a retention signal to ensure ER localization of Trx1 and TrxR1. A pCMV-ER/GFP-myc plasmid, containing an ER-targeted GFP within the exact same cloning site, was used as a transfection control. The transfection was validated by examining the expressed proteins by Western blot analysis (Figure 6).

**Figure 7:**
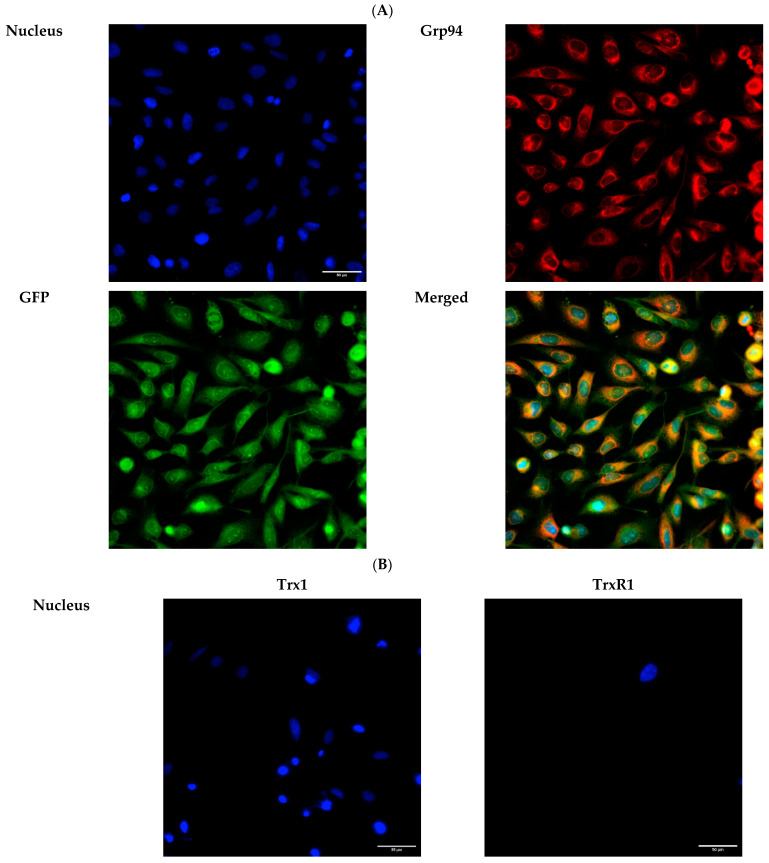

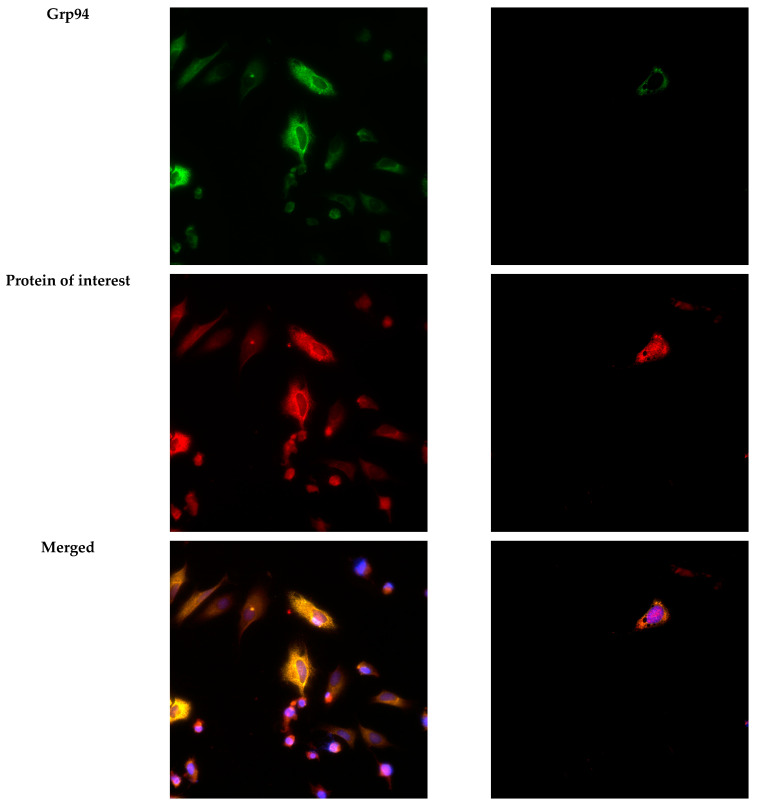
Immunofluorescence analysis of HeLa cells transfected with ER-targeted GFP, or Trx1 and TrxR1. (**A**) Immunofluorescence analysis of HeLa cells transfected with pCMV-ER/GFP-myc plasmid. GFP shows co-localization with the endoplasmic reticulum marker Grp94 (red). (**B**) Trx1 and TrxR1 (red) show co-localization with the ER marker Grp94 (green) in pCMV-ER/Trx1-myc and pCMV-ER/TrxR1-myc co-transfected HeLa cells. Cells were immunoreacted with antibodies against Trx1 or TrxR1 and the ER marker Grp94, as described in the Materials and Methods section. Images were acquired by fluorescence microscopy, as reported in the Materials and Methods section, at 40× magnification. Scalebar: 50 µm.

To verify the expression of GFP, Trx1, and TrxR1 proteins in the lumen of the ER, we performed immunofluorescence analysis 24 h post-transfection. The ER-targeted expression of GFP was verified by examining the co-localization of GFP and the ER marker Grp94 using immunofluorescence analysis in HeLa cells (Figure 7A). As expected, both transfected Trx1 and TrxR1 showed co-localization with the ER marker Grp94, confirming the luminal localization of the transfected proteins (Figure 7B).

**Figure 8:**
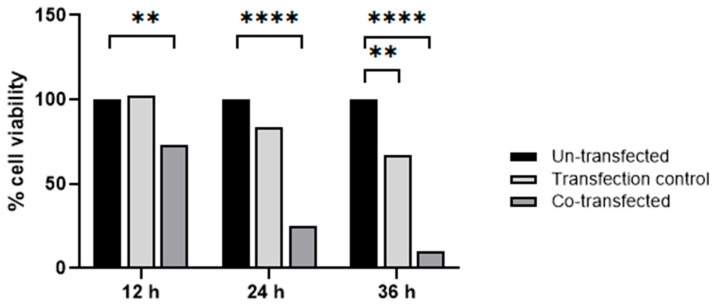
Viability of HeLa cells co-transfected with ER-targeted Trx1/TrxR1 compared to un-transfected control cells 12 h, 24 h, and 36 h post-transfection. The pCMV-ER/GFP-myc plasmid was used as transfection control; *p* values: ** = 0.01, **** ≤ 0.0001, Shapiro–Wilk test followed by Tukey’s multiple comparison test.

Next, we analyzed the cell viability of HeLa cells co-transfected with pCMV-ER/Trx1-myc and pCMV-ER/TrxR1-myc plasmids. The same plasmid with an ER-targeted GFP (pCMV-ER/GFP-myc plasmid) was used as a transfection control. Cell viability was measured at 12, 24, and 36 h post-transfection and was compared to the viability of un-transfected HeLa cells (Figure 8). At 12 h post-transfection, the decrease in cell viability of pCMV-ER/Trx1-myc and pCMV-ER/TrxR1-myc co-transfected cells was statistically significant, while transfection with pCMV-ER/GFP-myc did not affect viability. After 24 h, the viability of ER-targeted Trx1/TrxR1 co-transfected cells dropped below 30%, while there was still no significant change in the viability of pCMV-ER/GFP-myc transfected cells. At 36 h post-transfection, we could hardly detect viable HeLa cells among the ER-targeted Trx1/TrxR1 clones, and the viability of control transfected cells has started to decrease too. Taken together, these data indicate that the expression of Trx1 and TrxR1 in the ER causes a significant reduction in cell viability of HeLa cells.

**Figure 9:**
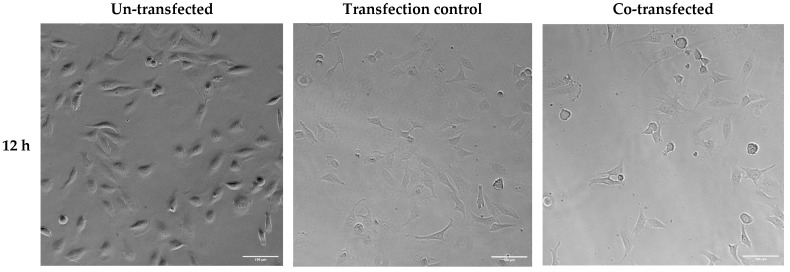

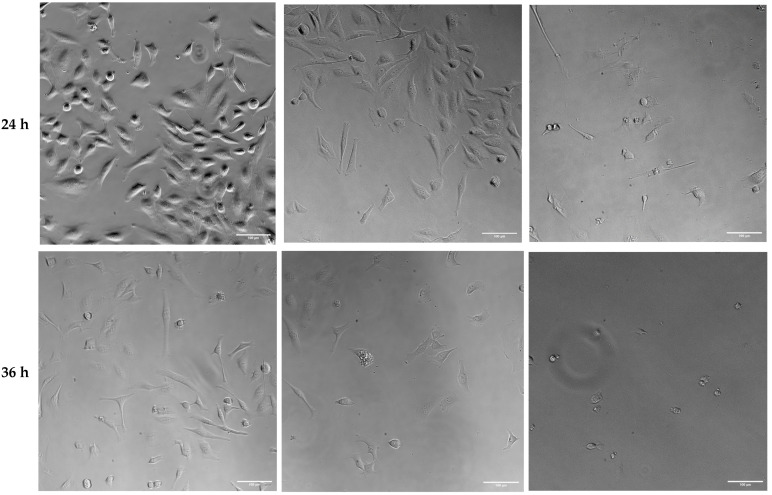
Brightfield analysis of HeLa cells transfected with pCMV-ER/GFP-myc plasmid (transfection control) or co-transfected with pCMV-ER/Trx1-myc and pCMV-ER/TrxR1-myc. The cell number was decreased, and the cell shape was altered in the co-transfected cells 24 and 36 h post-transfection. Images were acquired at 20× magnification. Scalebar: 100 µm.

Altered cell viability and morphology were also observed in brightfield images of HeLa cells 12, 24, and 36 h after transfection with ER-targeted Trx1/TrxR1 (Figure 9). Cell viability was visibly decreased, and cell morphology was also altered in the 24 h post-transfection samples.

**Figure 10:**
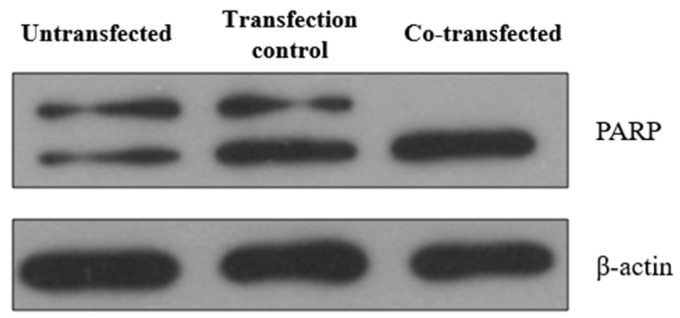
Co-transfection of HeLa cells with pCMV-ER/Trx1-myc and pCMV-ER/TrxR1-myc resulted in apoptotic cell death. PARP expression and cleavage were investigated by Western blot analysis after SDS-PAGE separation of proteins. Lysates of untreated HeLa cells, pCMV-ER/GFP-myc plasmid transfected cells, and pCMV-ER/Trx1-myc and pCMV-ER/TrxR1-myc co-transfected HeLa cells were analyzed. Equal amounts of protein were loaded in each lane (25 μg). β-actin was used as a loading control.

In order to confirm the apoptosis of HeLa cells 24 h after co-transfection with pCMV-ER/Trx1-myc and pCMV-ER/TrxR1-myc, we examined the cleavage of the 116 kDa Poly(ADP-ribose) Polymerase (PARP) by Western blot analysis (Figure 10). In the co-transfected cells, PARP cleavage to an 85 kDa fragment indicated the presence of apoptosis [42]. These results suggest that the expression of Trx1 and TrxR1 in the ER induces rapid apoptotic cell death in HeLa cells.

## 3. Discussion

The negligible representation of glutathione reductase in the ER was described previously [9]. However, there are only scarce data on the localization of the Trx/TrxR system. The localization of the TrxR protein was investigated almost 30 years ago by immunoblot analysis, comparing the protein expression of the ER and cytosolic fractions. It was found that TrxR protein was exclusively present in the cytosolic fraction [31]. The study had indisputable relevance and was used as an orientation in the majority of further investigations; we sought to assess a comprehensive investigation of the localization of the Trx/TrxR system including all major organelles, with special emphasis on the ER. In the current work, we investigated the expression and enzyme activity of all known isoforms of thioredoxins and thioredoxin reductases using various in silico and in vitro techniques. To demonstrate the presence or absence of these isoforms in a single cellular compartment, methods that are widely accepted and considered to be the most appropriate to this aim were used, such as microscopical analysis, database search, and protein expression studies [43].

Available databases, such as the Human Proteome Atlas (HPA, http://www.proteinatlas.org/, [44], accessed on 23 April 2024), can be a useful tool for assessing the intracellular localization of proteins, but they usually have limitations and are not a substitute for more detailed localization studies. The HPA assesses the subcellular distribution of proteins solely by immunocytochemistry/immunofluorescence analysis, and human data for Trx/TrxR isoforms are exclusively derived from cancer cell lines. Furthermore, not all Trx/TrxR isoform localizations are confirmed by the HPA and fall into only a lower reliability category. In our manuscript, we have also tested protein expression by Western blot analysis and enzyme activity on subcellular fractions, and not only on cancer cell lines, but also on healthy human fibroblasts. Importantly, our results were not always consistent with the HPA’s data. For Trx1 and Trx2, we observed localizations similar to HPA; however, HPA localizes TrxR1 to the nucleoplasm, whereas our findings indicated a predominantly cytosolic localization, which is in accordance with the majority of the literature [16,45,46]). In the case of TrxR2, we observed a mitochondrial localization in accordance with the HPA; however, the reliability of the localization fell only into a lower category. The HPA localizes TrxR3 mainly to the nucleoplasm, in addition to the cytosol, while our experiments mainly show cytosolic location of the protein.

Besides the HPA, several databases provide information on the subcellular localization of Trx and TrxR isoforms. For instance, the Map of the Cell (http://mapofthecell.biochem.mpg.de/, [47], accessed on 27 May 2024) provides quantitative information on the proteome of HeLa cells, including intracellular distribution; however, the organellar localization of TrxR3 is unassigned. ProLocate (https://prolocate.cabm.rutgers.edu/index.cgi, [48], accessed on 27 May 2024) provides information on the location of over 6000 components of the rat liver proteome, but there are no data on TrxR3. The Compartments database (https://compartments.jensenlab.org/Search, [49], accessed on 27 May 2024) integrates evidence for protein subcellular localization from several different sources, including manually curated literature, high-throughput screens, automatic text mining, and sequence-based prediction methods, and then assigns a confidence score to each. Interestingly, the ER localization of each isoform was identified in the text mining section of Compartments, although with only low or moderate confidence.

Our in silico analysis with eight different prediction tools equivocally indicated that the presence of human Trx1, Trx2, TrxR1, TrxR2, and TrxR3 in the ER lumen is highly unlikely. Next, the specific enzyme activity of TrxR was measured on rat liver subcellular fractions; as expected, the highest thioredoxin reductase activity was detected in the cytoplasmic and mitochondrial fractions: the former mainly occurred due to the TrxR1, the latter due to TrxR2 expression [15,16,40,50], and the activity was virtually negligible in the microsomal fraction. Therefore, it can be indisputably concluded that the absence of TrxR in the ER lumen is highly certain, and this fact evidently supports the presumption of uncoupled state of the redox systems in the ER lumen. In addition, our Western blot experiments also showed the absence of the three isoforms of TrxR and two isoforms of Trx protein from the ER-derived microsomal fraction. In the case of Trx1 and TrxR2, a faint band might be observed in the ER-derived fraction, which can be attributed to the presence of ER contact sites with other organelles and the inadequacy of the fractionation techniques to separate these. Our results confirmed that the highest TrxR1 expression was found in the cytoplasm, and the TrxR2 expression was connected to the mitochondrial fraction. These results were in accordance with the literature, as mammalian TrxR1 expression analysis mainly showed cytoplasmic localization, while TrxR2 was shown to be expressed in the mitochondria and possesses a mitochondrial localization signal [15,16,40,50]. The exact localization of TrxR3 is currently uncertain in the databases, but it seems to be localized in the cytoplasm based on our Western blot results. Ultimately, the determination of the intracellular location of TrxRs was achieved also by immunofluorescent analysis of HeLa and fibroblast cell lines. Finally, none of the microscopic images showed any Trx or TrxR co-localization with the specific ER markers, and the cytosolic location of TrxR3 was confirmed in these experiments. So far, the expression of TrxR3 was shown in different tissues, such as testis, cardiovascular tissues, and the colon, and we cannot exclude that the protein may show a different subcellular distribution in different tissue types.

In order to study how the disturbance of the physiological uncoupling of the reduced pyridine nucleotide system and the oxidized proteins can alter cellular homeostasis, we generated an ER-targeted version of Trx1 and TrxR1. Unfortunately, overexpressing selenoproteins in mammalian cells poses a significant challenge due to the complex and inefficient selenoprotein synthesis machinery. Attempts to overexpress TrxR proteins typically result in a mixture of mostly UGA-truncated proteins, and a small fraction of full-length selenocysteine-containing enzymes. The presence of selenocysteine-deficient TrxR variants potentially leads to cell death due to their prooxidant properties [51]. This may be the reason why the generation of cell lines stably overexpressing TrxR1 in Jurkat, HeLa, and U1285 cells was unsuccessful so far in the literature [52]. We also failed to generate stable expressing cell lines, because transient transfection of the ER-targeted members of the Trx1/TrxR1 system into HeLa cells resulted in rapid apoptotic cell death. This might suggest that coupling the two separated redox systems in the ER lumen can be detrimental to cellular viability.

Despite its absence from the lumen, a contribution of the Trx/TrxR system to luminal reduction of misfolded proteins has recently been suggested: a cytosolically located Trx system was shown to transfer reducing agents from NADPH via a yet-unknown membrane protein towards the lumen to reduce incorrectly folded protein disulfides [53]. These data further support the fact that intraluminal pathways connecting NADPH and protein thiol/disulfide systems are not essential for cell survival. Furthermore, intraluminal connection of the two systems might have deleterious consequences, as proper disulfide formation and oxidative capacity of the lumen would be compromised.

To date, the presence of Trx/TrxR systems in the ER remains a limitedly investigated area. Our study provides the first comprehensive analysis of the localization and activity of Trx/TrxR proteins, with special emphasis on their presence in the ER lumen. The relevance of the results lies in the fact that in silico predictions were confirmed by various in vitro experiments, where we proved the lack of the three human isoforms of TrxR (TrxR1, 2, 3) and two isoforms of Trx (Trx1, 2) and showed that their activity and the expression are absent from rat liver ER lumen.

## 4. Materials and Methods

### 4.1. In Silico Analysis

The following in silico prediction tools were used to predict the subcellular localization of the proteins of interest: PSORT II (https://psort.hgc.jp/form2.html) [32], Predotar (https://urgi.versailles.inra.fr/predotar/predotar.html) [33], Cello v.2.5 (http://cello.life.nctu.edu.tw/) [34], MultiLoc2 (https://github.com/KohlbacherLab/MultiLoc2) [35], yLoc (https://github.com/KohlbacherLab/YLoc) [36], LocTree3 (https://github.com/Rostlab/LocTree3) [37], and DeepLoc-1.0 (https://services.healthtech.dtu.dk/services/DeepLoc-1.0/) [38], all accessed on 8 August 2023. Amino acid sequences were obtained from the UniProt database (15.11.2021): Trx1 (Uniprot ID: P10599), Trx2 (Uniprot ID: Q99757), TrxR1 (Uniprot ID: Q16881), TrxR2 (Uniprot ID: Q9NNW7), TrxR3 (Uniprot ID: Q86VQ6). The amino acid sequences of Trx and TrxR isoforms are available in Table S1.

### 4.2. Animals

Male Wistar rats (180–230 g, Charles River Europe Laboratories Inc. Toxi-Coop Ltd., Budapest, Hungary) were kept with ad libitum access to food and water until used.

### 4.3. Preparation of Subcellular Fractions from Rat Liver Tissue

Subcellular fractions were prepared from overnight-fasted male Wistar rats by differential centrifugation, as previously reported, with slight modifications [54,55]. Briefly, freshly removed liver was cut into pieces and homogenized in sucrose-HEPES buffer (0.3 M of sucrose and 0.02 M of HEPES, pH 7.2) with a Potter–Elvehjem homogenizer. Homogenates were diluted to 20% with the same sucrose-HEPES buffer and centrifuged at 50× *g* for 1 h at 4 °C to remove cell debris. The supernatant was further centrifuged at 1000× *g* for 10 min at 4 °C. Mitochondrial fraction was obtained after centrifugation of the post-nuclear pellet (11,000× *g*, 20 min at 4 °C). The supernatant was ultracentrifuged (100,000× *g*, 60 min, 4 °C) to separate the cytosolic and microsomal fractions representing the endoplasmic reticulum. Finally, mitochondrial and microsomal fractions were resuspended in MOPS buffer (100 mM of KCl, 20 mM of NaCl, 1 mM of MgCl_2_, 20 mM of MOPS, pH 7.0), and all fractions were stored in liquid nitrogen until use. Protein concentrations of the fractions were determined using the Pierce BCA (Bicinchoninic Acid) Protein Assay Kit (Thermo Fisher Scientific Inc., Waltham, MA, USA; #23225) using bovine serum albumin as a standard, according to the manufacturer’s instructions.

### 4.4. Measurement of Thioredoxin Reductase Activity

To measure the activity of thioredoxin reductase, the Thioredoxin Reductase Assay Kit (Abcam, Cambridge, UK; ab83463) was used, according to the manufacturer’s instructions. The measurements were performed in a 96-well plate with 200 µg of protein per well derived from the rat liver fractions.

### 4.5. Sodium Dodecyl Sulphate Polyacrilamide Gel Electrophoresis (SDS-PAGE) and Western Blot Analysis

Equal amounts of total protein of subcellular fractions (25 µg in each) were run on 10 or 12% SDS polyacrylamide gel, as previously reported [56]. Proteins were electroblotted onto a PVDF or nitrocellulose membrane, blocked in 0.05% TBS-Tween containing 5% non-fat milk, and incubated with the primary antibody overnight at 4 °C, in 0.05% TBS-Tween containing 1% non-fat milk. After washing steps (3 times in 0.05% TBS-Tween for 5 min), the membrane was incubated with the secondary antibody for 1 h in 0.05% TBS-Tween containing 1% non-fat milk. SuperSignal™ West Pico PLUS Chemiluminescent Substrate reagent (Thermo Fisher) was used for the visualization.

Organelle-specific marker antibodies were used to check the purity of the fractionation: GAPDH (Santa Cruz Biotechnology, Inc., Dallas, TX, USA; sc-47724) for cytosol, Cyclophilin D (MitoSciences, Eugene, OR, USA; MSA04) for mitochondria, and Grp94 (Abcam; ab212034) for ER, respectively.

Antibodies used in Western blot analysis of thioredoxins and thioredoxin reductases: Anti Trx1 (Cell Signaling, Cell Signaling Technology, Inc., Danvers, MA, USA; #2298S), Anti TrxR1 (Abcam; ab167411), Anti Trx2 (Cell Signaling; #13322S), Anti TrxR3 (Biorbyt Ltd., Cambridge, UK; orb326510). Antibodies used in the Western blot analysis of pCMV-Trx1-myc/pCMV-TrxR1-myc co-transfected HeLa cells: Anti PARP (Cell Signaling, #9532), Anti β-actin (Abcam, ab6276) and Anti c-myc (Invitrogen, Waltham, MA, USA; #MA1-980).

### 4.6. Cell Culture and Maintenance

hTERT-immortalized human fibroblast cells were cultured in vitro in Dulbecco’s Modified Eagle Medium (Life Technologies, Carlsbad, CA, USA) supplemented with 10% fetal bovine serum (Life Technologies), 1% minimum essential medium with non-essential amino acids (Life Technologies), and 1% penicillin–streptomycin (Life Technologies). HeLa cells were maintained in Eagle’s Minimum Essential Medium (Life Technologies) supplemented with 10% fetal bovine serum (Life Technologies) and 1% penicillin–streptomycin (Life Technologies). The cell cultures were incubated in humidified incubators at 37 °C in 95% air and 5% CO_2_.

### 4.7. Immunofluorescent Analysis of Human Fibroblast and HeLa Cells

For microscopic determination of the localization of Trx/TrxR isoforms, a 12 mm coverslip was placed in a 24-well plate, and 30,000 fibroblast or 50,000 HeLa cells were plated per well. Cells were counted with a Bürker chamber, traditionally. After overnight incubation at 37 °C in 95% air and 5% CO_2_, cells were washed once with PBS (pH 7.4), fixed on the coverslip with 100% ice-cold methanol for 20 min, and washed with PBS three times for 5 min. Cells were blocked by 0.05% PBS-Tween containing 1% bovine serum albumin (Sigma-Aldrich, St. Louis, MO, USA; A9647) and 5% goat serum (Themo Fisher, 5062Z) for 30 min and incubated overnight at 4 °C with the primary antibody. The following day, after washing with 0.05% PBS-Tween, cells were incubated with the secondary antibody in the dark (Invitrogen, anti-rabbit Alexa Fluor 568, #A-11011; anti-rat Alexa Fluor 488, #A-11006; anti-mouse Alexa Fluor 647, #A32728; or anti-rat Alexa Fluor 568, #A-11077 in case of the transient transfection of HeLa cells; all 1:500) for 1 h at room temperature. After the re-blocking step, an additional primary antibody was applied (Grp94 rat monoclonal; diluted 1:200 in 0.05% PBS-Tween or tubulin mouse monoclonal; diluted 1:1000 in 0.05% PBS-Tween). Cells were then washed with 0.05% PBS-Tween and incubated with the corresponding secondary antibody with conjugated fluorophore for 1 h at room temperature. After washing three times with PBS, the coverslip was embedded on a slide with ProLong Glass Antifade Mountant with DAPI (Invitrogen, #P36935). After drying in the dark for 24 h, the fluorescent signal was recorded with a Nikon Eclipse Ti2 inverted microscope (Nikon Instruments, Melville, NY, USA) equipped with 10×, 20×, 40×, and 60× oil immersion objective (Plan Apo lambda, N.A. 1.4) and a cooled sCMOS camera (Zyla 4.2, Andor Technology, Belfast, UK). Images were analyzed with ImageJ Version 1.54.

### 4.8. Brightfield Microscopy of HeLa Cells

Brightfield microscopy was performed with a Nikon Eclipse Ti2 inverted microscope (Nikon Instruments, Melville, NY, USA) equipped with 10×, 20×, 40×, and 60× oil immersion objective (Plan Apo lambda, N.A. 1.4) and a cooled sCMOS camera (Zyla 4.2, Andor Technology, Belfast, UK). Images were analyzed with ImageJ.

### 4.9. Plasmid Design and Construction

To construct pCMV-ER/Trx1-myc and pCMV-ER/TrxR1-myc, the GFP sequences of the pCMV-ER/GFP-myc plasmid (Invitrogen) were replaced with full-length cDNA sequences of human *TXN* and *TXNRD1*, encoding Trx1 and TrxR1, respectively. cDNA was amplified by PCR using two oligonucleotides that contained *Pst*I upstream of the initiator methionine codon and *Not*I just before the termination codon. The specific primers used were the following: Trx1 sense (5′-3′): TTTCTGCAGATGGTGAAGCAGATCGAGAGC, Trx1 antisense (5′-3′): TTGCGGCCGCGACTAATTCATTAATGGTGGC; TrxR1 sense (5′-3′): TTTCTGCAGATGAACGGCCCTGAAGATCTT, antisense (5′-3′): TTGCGGCCGCACCTCAGCAGCCAGCCTG. After digestion with *Pst*I and *Not*I, the cDNAs were ligated to *Pst*I/*Not*I-digested pCMV-ER/GFP-myc. The plasmids encode ER-targeted proteins of Trx1 and TrxR1.

### 4.10. Transient Co-Transfection of HeLa Cells

HeLa cells were grown and treated on 96-well plates. Cells were co-transfected with pCMV-ER/Trx1-myc and pCMV-ER/TrxR1-myc plasmids using Lipofectamine 3000 (Invitrogen, # L3000008), according to the manufacturer’s instructions. For brightfield microscopy, HeLa cells were grown in 24-well plates and were transfected with the abovementioned plasmids as described previously.

### 4.11. Cell Viability Assay

The relative number of viable cells was calculated by Burker chambers. Cell viability was detected using CellTiter-Blue assay (Promega, Madison, WI, USA; #G8080). Cells were grown and treated on 96-well plates and were incubated with resazurin for 2 h at 37 °C. Fluorescence was measured at 560/590 nm. Three parallel measurements were carried out.

### 4.12. Statistical Analysis

Significant differences in TrxR activity were determined using unpaired *t*-test using GraphPad PRISM version 7.0 (San Diego, CA, USA). Generated *p* values are displayed as: ***: 0.0002, **** = <0.0001.

Significant differences in pCMV-ER/Trx1-myc and pCMV-ER/TrxR1-myc co-transfected HeLa cell viability were determined using the Shapiro–Wilk test followed by Tukey’s multiple comparison test using GraphPad PRISM version 7.0. Generated *p* values are displayed as: **: 0.01, **** = <0.0001.

## 5. Conclusions

The different redox potential of the two redox systems in the lumen (i.e., pyridine nucleotide and protein thiol/disulfide) is ensured by their uncoupling, since both thioredoxin reductase and glutathione reductase [9] are hardly detectable there. Coupling the two systems by ER-targeted expression of Trx1 and TrxR1 resulted in apoptotic cell death shortly after transfection, indicating that separated luminal redox systems are necessary for cellular viability. The exact mechanisms by which coupling of the two systems has deleterious consequences remain the subject of further studies.

## Figures and Tables

**Table 1:** In silico prediction of endoplasmic reticulum localization of Trx/TrxR isoforms. The sequences of Trx and TrxR isoforms were obtained from the Uniprot database (http://www.uniprot.org), accessed on 15 November 2021 (bold: canonical form; ^†^: computationally mapped). The amino acid sequences of Trx and TrxR isoforms are available in Table S1.

Isoform	PSORT II	Predotar	Cello	MultiLoc2	yLOC-HighRes	LocTree3	DeepLoc-1.0
**Trx1 isoform_1**	**-**	**1%**	**0.091**	**0%**	**0.0%**	**-**	**0.02%**
Trx1 isoform_2	-	1%	0.091	0%	0.0%	-	9.20%
**Trx2_isoform 1**	**-**	**1%**	**0.015**	**0%**	**0.0%**	**-**	**0.00%**
Trx2_isoform 2 ^†^	4.3%	-	0.031	0%	0.0%	-	0.19%
Trx2_isoform 3 ^†^	4.3%	1%	0.059	0%	0.0%	-	0.06%
TrxR1_isoform 1	-	1%	0.031	0%	0.0%	-	1.70%
TrxR1_isoform 2	-	1%	0.031	0%	0.0%	-	0.00%
TrxR1_isoform 3	-	1%	0.020	0%	0.0%	-	0.26%
TrxR1_isoform 4	-	1%	0.034	0%	0.0%	-	0.37%
**TrxR1_isoform 5**	**-**	**1%**	**0.030**	**0%**	**0.0%**	**-**	**1.25%**
TrxR1_isoform 6	-	6%	0.030	1%	1.6%	-	0.04%
TrxR1_isoform 7	-	1%	0.033	0%	0.0%	-	1.63%
TrxR1_isoform 8 ^†^	-	1%	0.022	1%	0.0%	-	0.00%
TrxR1_isoform 9 ^†^	-	1%	0.033	0%	0.0%	-	1.02%
TrxR1_isoform 10 ^†^	-	1%	0.034	0%	0.2%	-	1.78%
TrxR1_isoform 11 ^†^	-	1%	0.030	0%	0.0%	-	1.69%
TrxR1_isoform 12 ^†^	-	1%	0.038	0%	0.0%	-	0.70%
TrxR1_isoform 13 ^†^	-	1%	0.032	0%	0.0%	-	1.62%
**TrxR2_isoform 1**	**-**	**1%**	**0.026**	**0%**	**0.0%**	**-**	**0.00%**
TrxR2_isoform 2	-	1%	0.026	0%	0.0%	-	0.56%
TrxR2_isoform 3	-	1%	0.040	0%	0.0%	-	1.25%
TrxR2_isoform 4	-	1%	0.030	0%	0.0%	-	0.49%
TrxR2_isoform 5 ^†^	-	1%	0.026	0%	0.0%	-	2.11%
TrxR2_isoform 6 ^†^	-	1%	0.026	0%	0.0%	-	1.15%
TrxR2_isoform 7 ^†^	-	1%	0.025	0%	0.0%	-	0.00%
TrxR2_isoform 8 ^†^	-	1%	0.022	0%	0.0%	-	0.00%
**TrxR3_isoform 1**	**4.3%**	**1%**	**0.037**	**0%**	**0.0%**	**-**	**0.98%**
TrxR3_isoform 2 ^†^	4.3%	-	0.037	0%	0.0%	-	0.90%
TrxR3_isoform 3 ^†^	4.3%	1%	0.029	0%	0.0%	-	0.60%

**Table 2:** In silico prediction of the intracellular localization of TrxR3. The sequence of TrxR3 was obtained from the Uniprot database (http://www.uniprot.org), accessed on 15 November 2021.

Localization	PSORT II	Predotar	Cello	MultiLoc2	yLOC-HighRes	LocTree3	DeepLoc-1.0
Plasma membrane	-	-	0.122	0%	0.0%	-	1.79%
Endoplasmic reticulum	4.3%	1%	0.029	0%	0.0%	-	0.60%
Extracellular	-	-	0.253	0%	0.0%	-	1.70%
Lysosome	-	-	0.026	0%	0%	-	0.93%
Golgi	-	-	0.021	0%	0.0%	-	0.35%
Peroxysome	4.3%	-	0.271	30%	2.6%	-	16.43%
Mitochondria	17.4%	1%	1.401	10%	0.8%	-	11.35%
Cytosol	43.5%	-	1.248	58%	96.6%	100%	57.91%
Nucleus	21.7%	-	0.735	2%	0.0%	-	4.25%

## Data Availability

Dataset available upon request from the authors.

## Supplementary Materials

The supporting information can be downloaded at: https://www.mdpi.com/article/10.3390/ijms25126647/s1.
