# Supplementary material for: Subcellular Localization of Thioredoxin/Thioredoxin Reductase System—A Missing Link in Endoplasmic Reticulum Redox Balance
Source: Int J Mol Sci. 2024 Jun 17;25(12):6647. doi: 10.3390/ijms25126647 (PMC11204020; doi:10.3390/ijms25126647)
Supplement: Supplementary file 1 [file ijms-25-06647-s001.zip › ijms-2950433-supplementary.pdf]

Supplementary Table S1. Amino acid sequences of Trx/TrxR isoforms. The sequences of Trx and TrxR isoforms were obtained from the Uniprot database (http://www.uniprot.org) (bold: canonical form; †: computationally mapped)

Isoform	Amino acid sequence
**Trx1 isoform_1**	MVKQIESKTAFQEALDAAGDKLVVVDFSATWCGPCKMIKPFFHSLSEKYSNVIFLEVDVD<br>DCQDVASECEVKCMPTFQFFKKGQKVGEFSGANKEKLEATINELV
Trx1 isoform_2	MVKQIESKTAFQEALDAAGDKLVVVDFSATWCGPCKMIKPFFHDVASECEVKCMPTFQFF<br>KKGQKVGEFSGANKEKLEATINELV
**Trx2_isoform 1**	MAQRLLLRRFLASVISRKPSQGQWPPLTSRALQTPQCSPGGLTVTPNPARTIYTTRISLT<br>TFNIQDGPDFQDRVVNSETPVVVDFHAQWCGPCKILGPRLEKMVAKQHGKVVMAKVDIDD
Trx2_isoform 2 †	IEVYHSYFWPLEWTIPSRDNNKCYAKIICNTKDNERVVGFHVLGPNAGEVTQGFAAALKC<br>MAQRLLLRRF LASVISRKPS QGQWPPLTSR ALQTPQCSPG GLTVTPNPAR<br>TIYTTRISLT TFNIQDGPDF QDRVVNSETP VVVDFHAQWC GPCKILGPRL<br>EKMVAKQHGK VVMAKVDIDD HTDLAIEYEA GSLSPRLECS GTITAH
Trx2_isoform 3 †	MVAKQHGKVVMAKVDIDDHTDLAIEYEVSAVPTVLAMKNGDVVDKFVGIKDEDQLEAFLK<br>KLIG
TrxR1_isoform 1	MGCAEGKAVAAAAPTELQTKGKNGDGRRRSAKDHHPGKTLPENPAGFTSTATADSRALLQ<br>AYIDGHSVVIFSRSTCTRCTEVKKLFKSLCVPYFVLELDQTEDGRALEGTLSELAAETDL<br>PVVFVKQRKIGGHGPTLKAYQEGRLQKLLKMNGPEDLPKSYDYDLIIIGGGSGGLAAAKE<br>AAQYGKKVMVLDFVTPTPLGTRWGLGGTCVNVGCIPKKLMHQAALLGQALQDSRNYGWKV<br>EETVKHDWDRMIEAVQNHIGSLNWGYRVALREKKVVYENAYGQFIGPHRIKATNNKGKEK<br>IYSAERFLIATGERPRYLGIPGDKEYCISSDDLFSLPYCPGKTLVVGASYVALECAGFLA<br>GIGLDVTVMVRSILLRGFDQDMANKIGEHMEEHGIKFIRQFVPIKVEQIEAGTPGRLRVV<br>AQSTNSEEIIEGEYNTVMLAIGRDACTRKIGLETVGVKINEKTGKIPVTDEEQTNVPYIY<br>AIGDILEDKVELTPVAIQAGRLLAQRLYAGSTVKCDYENVPTTVFTPLEYGACGLSEEKA<br>VEKFGEENIEVYHSYFWPLEWTIPSRDNNKCYAKIICNTKDNERVVGFHVLGPNAGEVTQ<br>GFAAALKCGLTKKQLDSTIGIHPVCAEVFTTLSVTKRSGASILQAGCUG
TrxR1_isoform 2	MLSRLVLNSWAQAIIRPRPPKVLGLQVTTFSEAYQEGRLQKLLKMNGPEDLPKSYDYDLI<br>IIGGGSGGLAAAKEAAQYGKKVMVLDFVTPTPLGTRWGLGGTCVNVGCIPKKLMHQAALL<br>GQALQDSRNYGWKVEETVKHDWDRMIEAVQNHIGSLNWGYRVALREKKVVYENAYGQFIG<br>PHRIKATNNKGKEKIYSAERFLIATGERPRYLGIPGDKEYCISSDDLFSLPYCPGKTLVV<br>GASYVALECAGFLAGIGLDVTVMVRSILLRGFDQDMANKIGEHMEEHGIKFIRQFVPIKV<br>EQIEAGTPGRLRVVAQSTNSEEIIEGEYNTVMLAIGRDACTRKIGLETVGVKINEKTGKI

	PVTDEEQTNVPYIYAIGDILEDKVELTPVAIQAGRLLAQRLYAGSTVKCDYENVPTTVFT<br>PLEYGACGLSEEKAVEKFGEENIEVYHSYFWPLEWTIPSRDNNKCYAKIICNTKDNERVV<br>GFHVLGPNAGEVTQGFAAALKCGLTKKQLDSTIGIHPVCAEVFTTLSVTKRSGASILQAG<br>CUG
TrxR1_isoform 3	MQQVMLTCKGVNRGHAVPAGPGRKPRPRRSSRLLAGEKHLTRSALLLCHTEDGRALEGTL<br>SELAAETDLPVVFVKQRKIGGHGPTLKAYQEGRLQKLLKMNGPEDLPKSYDYDLIIIGGG<br>SGGLAAAKEAAQYGKKVMVLDFVTPTPLGTRWGLGGTCVNVGCIPKKLMHQAALLGQALQ<br>DSRNYGWKVEETVKHDWDRMIEAVQNHIGSLNWGYRVALREKKVVYENAYGQFIGPHRIK<br>ATNNKGKEKIYSAERFLIATGERPRYLGIPGDKEYCISSDDLFSLPYCPGKTLVVGASYV<br>ALECAGFLAGIGLDVTVMVRSILLRGFDQDMANKIGEHMEEHGIKFIRQFVPIKVEQIEA<br>GTPGRLRVVAQSTNSEEIIEGEYNTVMLAIGRDACTRKIGLETVGVKINEKTGKIPVTDE<br>EQTNVPYIYAIGDILEDKVELTPVAIQAGRLLAQRLYAGSTVKCDYENVPTTVFTPLEYG<br>ACGLSEEKAVEKFGEENIEVYHSYFWPLEWTIPSRDNNKCYAKIICNTKDNERVVGFHVL<br>GPNAGEVTQGFAAALKCGLTKKQLDSTIGIHPVCAEVFTTLSVTKRSGASILQAGCUG
TrxR1_isoform 4	MSCEDGRALEGTLSELAAETDLPVVFVKQRKIGGHGPTLKAYQEGRLQKLLKMNGPEDLP<br>KSYDYDLIIIGGGSGGLAAAKEAAQYGKKVMVLDFVTPTPLGTRWGLGGTCVNVGCIPKK<br>LMHQAALLGQALQDSRNYGWKVEETVKHDWDRMIEAVQNHIGSLNWGYRVALREKKVVYE<br>NAYGQFIGPHRIKATNNKGKEKIYSAERFLIATGERPRYLGIPGDKEYCISSDDLFSLPY<br>CPGKTLVVGASYVALECAGFLAGIGLDVTVMVRSILLRGFDQDMANKIGEHMEEHGIKFI<br>RQFVPIKVEQIEAGTPGRLRVVAQSTNSEEIIEGEYNTVMLAIGRDACTRKIGLETVGVK<br>INEKTGKIPVTDEEQTNVPYIYAIGDILEDKVELTPVAIQAGRLLAQRLYAGSTVKCDYE<br>NVPTTVFTPLEYGACGLSEEKAVEKFGEENIEVYHSYFWPLEWTIPSRDNNKCYAKIICN<br>TKDNERVVGFHVLGPNAGEVTQGFAAALKCGLTKKQLDSTIGIHPVCAEVFTTLSVTKRS<br>GASILQAGCUG
**TrxR1_isoform 5**	MNGPEDLPKSYDYDLIIIGGGSGGLAAAKEAAQYGKKVMVLDFVTPTPLGTRWGLGGTCV<br>NVGCIPKKLMHQAALLGQALQDSRNYGWKVEETVKHDWDRMIEAVQNHIGSLNWGYRVAL<br>REKKVVYENAYGQFIGPHRIKATNNKGKEKIYSAERFLIATGERPRYLGIPGDKEYCISS<br>DDLFSLPYCPGKTLVVGASYVALECAGFLAGIGLDVTVMVRSILLRGFDQDMANKIGEHM<br>EEHGIKFIRQFVPIKVEQIEAGTPGRLRVVAQSTNSEEIIEGEYNTVMLAIGRDACTRKI<br>GLETVGVKINEKTGKIPVTDEEQTNVPYIYAIGDILEDKVELTPVAIQAGRLLAQRLYAG<br>STVKCDYENVPTTVFTPLEYGACGLSEEKAVEKFGEENIEVYHSYFWPLEWTIPSRDNNK<br>CYAKIICNTKDNERVVGFHVLGPNAGEVTQGFAAALKCGLTKKQLDSTIGIHPVCAEVFT<br>TLSVTKRSGASILQAGCUG

TrxR1_isoform 6	MPVDDYWLCLPASCARPFVQTVRVVQSCPHCCWFPGVLPSVPEPLRMPAMLPTGSHSAVL<br>PPSHCSTAPPSTSQEPSSSADPKLCLSPPTSDSRQERNVQFGLAYQEGRLQKLLKMNGPE<br>DLPKSYDYDLIIIGGGSGGLAAAKEAAQYGKKVMVLDFVTPTPLGTRWGLGGTCVNVGCI<br>PKKLMHQAALLGQALQDSRNYGWKVEETVKHDWDRMIEAVQNHIGSLNWGYRVALREKKV<br>VYENAYGQFIGPHRIKATNNKGKEKIYSAERFLIATGERPRYLGIPGDKEYCISSDDLFS<br>LPYCPGKTLVVGASYVALECAGFLAGIGLDVTVMVRSILLRGFDQDMANKIGEHMEEHGI<br>KFIRQFVPIKVEQIEAGTPGRLRVVAQSTNSEEIIEGEYNTVMLAIGRDACTRKIGLETV<br>GVKINEKTGKIPVTDEEQTNVPYIYAIGDILEDKVELTPVAIQAGRLLAQRLYAGSTVKC<br>DYENVPTTVFTPLEYGACGLSEEKAVEKFGEENIEVYHSYFWPLEWTIPSRDNNKCYAKI<br>ICNTKDNERVVGFHVLGPNAGEVTQGFAAALKCGLTKKQLDSTIGIHPVCAEVFTTLSVT<br>KRSGASILQAGCUG
TrxR1_isoform 7	MVLDFVTPTPLGTRWGLGGTCVNVGCIPKKLMHQAALLGQALQDSRNYGWKVEETVKHDW<br>DRMIEAVQNHIGSLNWGYRVALREKKVVYENAYGQFIGPHRIKATNNKGKEKIYSAERFL<br>IATGERPRYLGIPGDKEYCISSDDLFSLPYCPGKTLVVGASYVALECAGFLAGIGLDVTV<br>MVRSILLRGFDQDMANKIGEHMEEHGIKFIRQFVPIKVEQIEAGTPGRLRVVAQSTNSEE<br>IIEGEYNTVMLAIGRDACTRKIGLETVGVKINEKTGKIPVTDEEQTNVPYIYAIGDILED<br>KVELTPVAIQAGRLLAQRLYAGSTVKCDYENVPTTVFTPLEYGACGLSEEKAVEKFGEEN<br>IEVYHSYFWPLEWTIPSRDNNKCYAKIICNTKDNERVVGFHVLGPNAGEVTQGFAAALKC<br>GLTKKQLDSTIGIHPVCAEVFTTLSVTKRSGASILQAGCUG
TrxR1_isoform 8 †	MPAMLPTGSHSAVLPPSHCSTAPPSTSQEPSSSADPKLCLSPPTSDSRQERNVQFGLAYQ<br>EGRLQKLLKMNGPEDLPKSYDYDLIIIGGGSGGLAAAKEAAQYGKKVMVLDFVTPTPLGT<br>RWGLGGTCVNVGCIPKKLMHQAALLGQALQDSRNYGWKVEETVKHDWDRMIEAVQNHIGS<br>LNWGYRVALREKKVVYENAYGQFIGPHRIKATNNKGKEKIYSAERFLIATGERPRYLGIP<br>GDKEYCISSDDLFSLPYCPGKTLVVGASYVALECAGFLAGIGLDVTVMVRSILLRGFDQD<br>MANKIGEHMEEHGIKFIRQFVPIKVEQIEAGTPGRLRVVAQSTNSEEIIEGEYNTVMLAI<br>GRDACTRKIGLETVGVKINEKTGKIPVTDEEQTNVPYIYAIGDILEDKVELTPVAIQAGR<br>LLAQRLYAGSTVKCDYENVPTTVFTPLEYGACGLSEEKAVEKFGEENIEVYHSYFWPLEW<br>TIPSRDNNKCYAKIICNTKDNERVVGFHVLGPNAGEVTQGFAAALKCGLTKKQLDSTIGI<br>HPVCAEVFTTLSVTKRSGASILQAGCCG
TrxR1_isoform 9 †	MEDGRALEGTLSELAAETDLPVVFVKQRKIGGHGPTLKAYQEGRLQKLLKMNGPEDLPKS<br>YDYDLIIIGGGSGGLAAAKEAAQYGKKVMVLDFVTPTPLGTRWGLGGTCVNVGCIPKKLM<br>HQAALLGQALQDSRNYGWKVEETVKHDWDRMIEAVQNHIGSLNWGYRVALREKKVVYENA<br>YGQFIGPHRIKATNNKGKEKIYSAERFLIATGERPRYLGIPGDKEYCISSDDLFSLPYCP

	GKTLVVGASYVALECAGFLAGIGLDVTVMVRSILLRGFDQDMANKIGEHMEEHGIKFIRQ<br>FVPIKVEQIEAGTPGRLRVVAQSTNSEEIIEGEYNTVMLAIGRDACTRKIGLETVGVKIN<br>EKTGKIPVTDEEQTNVPYIYAIGDILEDKVELTPVAIQAGRLLAQRLYAGSTVKCDYENV<br>PTTVFTPLEYGACGLSEEKAVEKFGEENIEVYHSYFWPLEWTIPSRDNNKCYAKIICNTK<br>DNERVVGFHVLGPNAGEVTQGFAAALKCGLTKKQLDSTIGIHPVCAEVFTTLSVTKRSGA<br>SILQAGCG
TrxR1_isoform 10 †	MVLDFVTPTPLGTRWGLGGTCVNVGCIPKKLMHQAALLGQALQDSRNYGWKVEETVKHDW<br>DRMIEAVQNHIGSLNWGYRVALREKKVVYENAYGQFIGPHRIKATNNKGKEKIYSAERFL<br>IATGERPRYLGIPGDKEYCISSDDLFSLPYCPGKTLVVGASYVALECAGFLAGIGLDVTV<br>MVRSILLRGFDQDMANKIGEHMEEHGIKFIRQFVPIKVEQIEAGTPGRLRVVAQSTNSEE<br>IIEGEYNTVMLAIGRDACTRKIGLETVGVKINEKTGKIPVTDEEQTNVPYIYAIGDILED<br>KVELTPVAIQAGRLLAQRLYAGSTVKCDYENVPTTVFTPLEYGACGLSEEKAVEKFGEEN<br>IEVYHSYFWPLEWTIPSRDNNKCYAKIICNTKDNERVVGFHVLGPNAGEVTQGFAAALKC<br>GLTKKQLDSTIGIHPVCAEVFTTLSVTKRSGASILQAGCG
TrxR1_isoform 11 †	MNGPEDLPKSYDYDLIIIGGGSGGLAAAKEAAQYGKKVMVLDFVTPTPLGTRWGLGGTCV<br>NVGCIPKKLMHQAALLGQALQDSRNYGWKVEETVKHDWDRMIEAVQNHIGSLNWGYRVAL<br>REKKVVYENAYGQFIGPHRIKATNNKGKEKIYSAERFLIATGERPRYLGIPGDKEYCISS<br>DDLFSLPYCPGKTLVVGASYVALECAGFLAGIGLDVTVMVRSILLRGFDQDMANKIGEHM<br>EEHGIKFIRQFVPIKVEQIEAGTPGRLRVVAQSTNSEEIIEGEYNTVMLAIGRDACTRKI<br>GLETVGVKINEKTGKIPVTDEEQTNVPYIYAIGDILEDKVELTPVAIQAGRLLAQRLYAG<br>STVKCDYENVPTTVFTPLEYGACGLSEEKAVEKFGEENIEVYHSYFWPLEWTIPSRDNNK<br>CYAKIICNTKDNERVVGFHVLGPNAGEVTQGFAAALKCGLTKKQLDSTIGIHPVCAEVFT<br>TLSVTKRSGASILQAGCG
TrxR1_isoform 12 †	MNGPEDLPKSYDYDLIIIGGGSGGLAAAKEAAQYGKKVMVLDFVTPTPLGTRWGLGGTCV<br>NVGCIPKKLMHQAALLGQALQDSRNYGWKVEETVKHDWDRMIEAVQNHIGSLNWGYRVAL<br>REKKVVYENAYGQFIGPHRIKATNNKGKEKIYSAERFLIATGERPRYLGIPGDKEYCISS<br>DDLFSLPYCPGKTLVVGASYVALECAGFLAGIGLDVTVMVRSILLRGFDQDMANKIGEHM<br>EEHGIKFIRQFVPIKVEQIEAGTPGRLRVVAQSTNSEEIIEGEYNTVMLAIGRDACTRKI<br>GLETVGVKINEKTGKIPVTDEEQTNVPYIYAIGDILEDKVELTPVAIQAGRLLAQRLYAG<br>STVKCDYENVPTTVFTPLEYGACGLSEEKAVEKFGEENIEVYHSYFWPLEWTIPSRDNNK<br>CYAKIICNTKDNERVVGFHVLGPNAGEVTQGFAAALKCGLTKKQLDSTIGIHPVCAEFFS<br>FI

TrxR1_isoform 13 †	MGCAEGKAVAAAAPTELQTKGKNGDGRRRSAKDHHPGKTLPENPAGFTSTATADSRALLQ<br>AYIDGHSVVIFSRSTCTRCTEVKKLFKSLCVPYFVLELDQTEDGRALEGTLSELAAETDL<br>PVVFVKQRKIGGHGPTLKAYQEGRLQKLLKMNGPEDLPKSYDYDLIIIGGGSGGLAAAKE<br>AAQYGKKVMVLDFVTPTPLGTRWGLGGTCVNVGCIPKKLMHQAALLGQALQDSRNYGWKV<br>EETVKHDWDRMIEAVQNHIGSLNWGYRVALREKKVVYENAYGQFIGPHRIKATNNKGKEK<br>IYSAERFLIATGERPRYLGIPGDKEYCISSDDLFSLPYCPGKTLVVGASYVALECAGFLA<br>GIGLDVTVMVRSILLRGFDQDMANKIGEHMEEHGIKFIRQFVPIKVEQIEAGTPGRLRVV<br>AQSTNSEEIIEGEYNTVMLAIGRDACTRKIGLETVGVKINEKTGKIPVTDEEQTNVPYIY<br>AIGDILEDKVELTPVAIQAGRLLAQRLYAGSTVKCDYENVPTTVFTPLEYGACGLSEEKA<br>VEKFGEENIEVYHSYFWPLEWTIPSRDNNKCYAKIICNTKDNERVVGFHVLGPNAGEVTQ<br>GFAAALKCGLTKKQLDSTIGIHPVCAEVFTTLSVTKRSGASILQAGCG
**TrxR2_isoform 1**	MAAMAVALRGLGGRFRWRTQAVAGGVRGAARGAAAGQRDYDLLVVGGGSGGLACAKEAAQ<br>LGRKVAVVDYVEPSPQGTRWGLGGTCVNVGCIPKKLMHQAALLGGLIQDAPNYGWEVAQP<br>VPHDWRKMAEAVQNHVKSLNWGHRVQLQDRKVKYFNIKASFVDEHTVCGVAKGGKEILLS<br>ADHIIIATGGRPRYPTHIEGALEYGITSDDIFWLKESPGKTLVVGASYVALECAGFLTGI<br>GLDTTIMMRSIPLRGFDQQMSSMVIEHMASHGTRFLRGCAPSRVRRLPDGQLQVTWEDST<br>TGKEDTGTFDTVLWAIGRVPDTRSLNLEKAGVDTSPDTQKILVDSREATSVPHIYAIGDV<br>VEGRPELTPIAIMAGRLLVQRLFGGSSDLMDYDNVPTTVFTPLEYGCVGLSEEEAVARHG<br>QEHVEVYHAHYKPLEFTVAGRDASQCYVKMVCLREPPQLVLGLHFLGPNAGEVTQGFALG<br>IKCGASYAQVMRTVGIHPTCSEEVVKLRISKRSGLDPTVTGCUG
TrxR2_isoform 2	MEDQRGAAAGQRDYDLLVVGGGSGGLACAKEAAQLGRKVAVVDYVEPSPQGTRWGLGGTC<br>VNVGCIPKKLMHQAALLGGLIQDAPNYGWEVAQPVPHDWRKMAEAVQNHVKSLNWGHRVQ<br>LQDRKVKYFNIKASFVDEHTVCGVAKGGKEILLSADHIIIATGGRPRYPTHIEGALEYGI<br>TSDDIFWLKESPGKTLVVGASYVALECAGFLTGIGLDTTIMMRSIPLRGFDQQMSSMVIE<br>HMASHGTRFLRGCAPSRVRRLPDGQLQVTWEDSTTGKEDTGTFDTVLWAIGRVPDTRSLN<br>LEKAGVDTSPDTQKILVDSREATSVPHIYAIGDVVEGRPELTPIAIMAGRLLVQRLFGGS<br>SDLMDYDNVPTTVFTPLEYGCVGLSEEEAVARHGQEHVEVYHAHYKPLEFTVAGRDASQC<br>YVKMVCLREPPQLVLGLHFLGPNAGEVTQGFALGIKCGASYAQVMRTVGIHPTCSEEVVK<br>LRISKRSGLDPTVTGCUG
TrxR2_isoform 3	MHQAALLGGLIQDAPNYGWEVAQPVPHDWRKMAEAVQNHVKSLNWGHRVQLQDRKVKYFN<br>IKASFVDEHTVCGVAKGGKEILLSADHIIIATGGRPRYPTHIEGALEYGITSDDIFWLKE<br>SPGKTLVVGASYVALECAGFLTGIGLDTTIMMRSIPLRGFDQQMSSMVIEHMASHGTRFL

	RGCAPSRVRRLPDGQLQVTWEDSTTGKEDTGTFDTVLWAIGRVPDTRSLNLEKAGVDTSP<br>DTQKILVDSREATSVPHIYAIGDVVEGRPELTPIAIMAGRLLVQRLFGGSSDLMDYDNVP<br>TTVFTPLEYGCVGLSEEEAVARHGQEHVEVYHAHYKPLEFTVAGRDASQCYVKMVCLREP<br>PQLVLGLHFLGPNAGEVTQGFALGIKCGASYAQVMRTVGIHPTCSEEVVKLRISKRSGLD<br>PTVTGCUG
TrxR2_isoform 4	MRSIPLRGFDQQMSSMVIEHMASHGTRFLRGCAPSRVRRLPDGQLQVTWEDSTTGKEDTG<br>TFDTVLWAIGRVPDTRSLNLEKAGVDTSPDTQKILVDSREATSVPHIYAIGDVVEGRPEL<br>TPIAIMAGRLLVQRLFGGSSDLMDYDNVPTTVFTPLEYGCVGLSEEEAVARHGQEHVEVY<br>HAHYKPLEFTVAGRDASQCYVKMVCLREPPQLVLGLHFLGPNAGEVTQGFALGIKCGASY<br>AQVMRTVGIHPTCSEEVVKLRISKRSGLDPTVTGCUG
TrxR2_isoform 5 †	MAAMAVALRGLGGRFRWRTQAVAGGVRGAARGAAAAQLGRKVAVVDYVEPSPQGTRWGLG<br>GTCVNVGCIPKKLMHQAALLGGLIQDAPNYGWEVAQPVPHDWRKMAEAVQNHVKSLNWGH<br>RVQLQDRKVKYFNIKASFVDEHTVCGVAKGGKEILLSADHIIIATGGRPRYPTHIEGALE<br>YGITSDDIFWLKESPGKTLVVGASYVALECAGFLTGIGLDTTIMMRSIPLRGFDQQMSSM<br>VIEHMASHGTRFLRGCAPSRVRRLPDGQLQVTWEDSTTGKEDTGTFDTVLWAIGRVPDTR<br>SLNLEKAGVDTSPDTQKILVDSREATSVPHIYAIGDVVEGRPELTPIAIMAGRLLVQRLF<br>GGSSDLMDYDNVPTTVFTPLEYGCVGLSEEEAVARHGQEHVEVYHAHYKPLEFTVAGRDA<br>SQCYVKMVCLREPPQLVLGLHFLGPNAGEVTQGFALGIKCGASYAQVMRTVGIHPTCSEE<br>VVKLRISKRSGLDPTVTGCCG
TrxR2_isoform 6 †	MEDQAGQRDYDLLVVGGGSGGLACAKEAAQLGRKVAVVDYVEPSPQGTRWGLGGTCVNVG<br>CIPKKLMHQAALLGGLIQDAPNYGWEVAQPVPHDWRKMAEAVQNHVKSLNWGHRVQLQDR<br>KVKYFNIKASFVDEHTVCGVAKGGKEILLSADHIIIATGGRPRYPTHIEGALEYGITSDD<br>IFWLKESPGKTLVVGASYVALECAGFLTGIGLDTTIMMRSIPLRGFDQQMSSMVIEHMAS<br>HGTRFLRGCAPSRVRRLPDGQLQVTWEDSTTGKEDTGTFDTVLWAIGRVPDTRSLNLEKA<br>GVDTSPDTQKILVDSREATSVPHIYAIGDVVEGRPELTPIAIMAGRLLVQRLFGGSSDLM<br>DYDNVPTTVFTPLEYGCVGLSEEEAVARHGQEHVEVYHAHYKPLEFTVAGRDASQCYVKM<br>VCLREPPQLVLGLHFLGPNAGEVTQGFALGIKCGASYAQVMRTVGIHPTCSEEVVKLRIS<br>KRSGLDPTVTGCCG
TrxR2_isoform 7 †	MAAMAVALRGLGGRFRWRTQAVAGGVRGAARGAAGQRDYDLLVVGGGSGGLACAKEAAQL<br>GRKVAVVDYVEPSPQGTRWGLGGTCVNVGCIPKKLMHQAALLGGLIQDAPNYGWEVAQPV<br>PHDWRKMAEAVQNHVKSLNWGHRVQLQDRKVKYFNIKASFVDEHTVCGVAKGGKEILLSA<br>DHIIIATGGRPRYPTHIEGALEYGITSDDIFWLKESPGKTLVVGASYVALECAGFLTGIG<br>LDTTIMMRSIPLRGFDQQMSSMVIEHMASHGTRFLRGCAPSRVRRLPDGQLQVTWEDSTT

	GKEDTGTFDTVLWAIGRVPDTRSLNLEKAGVDTSPDTQKILVDSREATSVPHIYAIGDVV<br>EGRPELTPIAIMAGRLLVQRLFGGSSDLMDYDNVPTTVFTPLEYGCVGLSEEEAVARHGQ<br>EHVEVYHAHYKPLEFTVAGRDASQCYVKMVCLREPPQLVLGLHFLGPNAGEVTQGFALGI<br>KCGASYAQVMRTVGIHPTCSEEVVKLRISKRSGLDPTVTGCCG
TrxR2_isoform 8 †	MAAMAVALRGLGGRFRWRTQAVAGGVRGAARGAAAGQRDYDLLVVGGGSGGLACAKEGTR<br>WGLGGTCVNVGCIPKKLMHQAALLGGLIQDAPNYGWEVAQPVPHDWRKMAEAVQNHVKSL<br>NWGHRVQLQDRKVKYFNIKASFVDEHTVCGVAKGGKEILLSADHIIIATGGRPRYPTHIE<br>GALEYGITSDDIFWLKESPGKTLVVGASYVALECAGFLTGIGLDTTIMMRSIPLRGFDQQ<br>MSSMVIEHMASHGTRFLRGCAPSRVRRLPDGQLQVTWEDSTTGKEDTGTFDTVLWAIGRV<br>PDTRSLNLEKAGVDTSPDTQKILVDSREATSVPHIYAIGDVVEGRPELTPIAIMAGRLLV<br>QRLFGGSSDLMDYDNVPTTVFTPLEYGCVGLSEEEAVARHGQEHVEVYHAHYKPLEFTVA<br>GRDASQCYVKMVCLREPPQLVLGLHFLGPNAGEVTQGFALGIK
**TrxR3_isoform 1**	MERSPPQSPGPGKAGDAPNRRSGHVRGARVLSPPGRRARLSSPGPSRSSEAREELRRHLV<br>GLIERSRVVIFSKSYCPHSTRVKELFSSLGVECNVLELDQVDDGARVQEVLSEITNQKTV<br>PNIFVNKVHVGGCDQTFQAYQSGLLQKLLQEDLAYDYDLIIIGGGSGGLSCAKEAAILGK<br>KVMVLDFVVPSPQGTSWGLGGTCVNVGCIPKKLMHQAALLGQALCDSRKFGWEYNQQVRH<br>NWETMTKAIQNHISSLNWGYRLSLREKAVAYVNSYGEFVEHHKIKATNKKGQETYYTAAQ<br>FVIATGERPRYLGIQGDKEYCITSDDLFSLPYCPGKTLVVGASYVALECAGFLAGFGLDV<br>TVMVRSILLRGFDQEMAEKVGSYMEQHGVKFLRKFIPVMVQQLEKGSPGKLKVLAKSTEG<br>TETIEGVYNTVLLAIGRDSCTRKIGLEKIGVKINEKSGKIPVNDVEQTNVPYVYAVGDIL<br>EDKPELTPVAIQSGKLLAQRLFGASLEKCDYINVPTTVFTPLEYGCCGLSEEKAIEVYKK<br>ENLEIYHTLFWPLEWTVAGRENNTCYAKIICNKFDHDRVIGFHILGPNAGEVTQGFAAAM<br>KCGLTKQLLDDTIGIHPTCGEVFTTLEITKSSGLDITQKGCUG
TrxR3_isoform 2 †	LERSPPQSPGPGKAGDAPNRRSGHVRGARVLSPPGRRARLSSPGPSRSSEAREELRRHLV<br>GLIERSRVVIFSKSYCPHSTRVKELFSSLGVECNVLELDQVDDGARVQEVLSEITNQKTV<br>PNIFVNKVHVGGCDQTFQAYQSGLLQKLLQEDLAYDYDLIIIGGGSGGLSCAKEAAILGK<br>KVMVLDFVVPSPQGTSWGLGGTCVNVGCIPKKLMHQAALLGQALCDSRKFGWEYNQQVRH<br>NWETMTKAIQNHISSLNWGYRLSLREKAVAYVNSYGEFVEHHKIKATNKKGQETYYTAAQ<br>FVIATGERPRYLGIQGDKEYCITSDDLFSLPYCPGKTLVVGASYVALECAGFLAGFGLDV<br>TVMVRSILLRGFDQEMAEKVGSYMEQHGVKFLRKFIPVMVQQLEKGSPGKLKVLAKSTEG<br>TETIEGVYNTVLLAIGRDSCTRKIGLEKIGVKINEKSGKIPVNDVEQTNVPYVYAVGDIL<br>EDKPELTPVAIQSGKLLAQRLFGASLEKCDYINVPTTVFTPLEYGCCGLSEEKAIEVYKK<br>ENLEIYHTLFWPLEWTVAGRENNTCYAKIICNKFDHDRVIGFHILGPNAGEVTQGFAAAM

	KCGLTKQLLDDTIGIHPTCGEVFTTLEITKSSGLDITQKGCCG
TrxR3_isoform 3 †	MERSPPQSPGPGKAGDAPNRRSGHVRGARVLSPPGRRARLSSPGPSRSSEAREELRRHLV<br>GLIERSRVVIFSKSYCPHSTRVKELFSSLGVECNVLELDQVDDGARVQEVLSEITNQKTV<br>PNIFVNKVHVGGCDQTFQAYQSGLLQKLLQEDLAYDYDLIIIGGGSGGLSCAKEAAILGK<br>KVMVLDFVVPSPQGTSWGLGGTCVNVGCIPKKLMHQAALLGQALCDSRKFGWEYNQQVRH<br>NWETMTKAIQNHISSLNWGYRLSLREKAVAYVNSYGEFVEHHKIKATNKKGQETYYTAAQ<br>FVIATGERPRYLGIQGDKEYCITSDDLFSLPYCPGKTLVVGASYVALECAGFLAGFGLDV<br>TVMVRSILLRGFDQEMAEKVGSYMEQHGVKFLRKFIPVMVQQLEKGSPGKLKVLAKSTEG<br>TETIEGVYNTVLLAIGRDSCTRKIGLEKIGVKINEKSGKIPVNDVEQTNVPYVYAVGDIL<br>EDKPELTPVAIQSGKLLAQRLFGASLEKIYHTLFWPLEWTVAGRENNTCYAKIICNKFDH<br>DRVIGFHILGPNAGEVTQGFAAAMKCGLTKQLLDDTIGIHPTCGEVFTTLEITKSSGLDI<br>TQKGCCG
